# Resampling QTL Effects in the QTL Sign Test Leads to Incongruous Sensitivity to Variance in Effect Size

**DOI:** 10.1534/g3.112.003228

**Published:** 2012-08-01

**Authors:** Daniel P. Rice, Jeffrey P. Townsend

**Affiliations:** * Department of Ecology and Evolutionary Biology, Yale University, New Haven, CT 06520; † Program in Computational Biology and Bioinformatics, Yale University, New Haven, CT 06520

**Keywords:** phenotype, selection, statistical tests, quantitative trait loci (QTL), neutrality

## Abstract

Allelic effects at quantitative trait loci (QTL) between lineages are potentially informative for indicating the action of natural selection. The QTL Sign Test uses the number of + and − alleles observed in a QTL study to infer a history of selection. This test has been constructed to condition on the phenotypic difference between the two lines in question. By applying the test to QTL data simulated under selection, we demonstrate that conditioning on the phenotypic difference results in a loss of power to reject the neutral hypothesis and marked sensitivity to variation in locus effect magnitude.

Distinguishing traits subject to selection from traits evolving neutrally is a challenging and important biological problem (Boake *et al.* 2002). It has been difficult to link the microevolutionary forces studied in contemporary populations to inferences about historical phenotypic selection (Grant and Grant 2002), yet this task is central to evolutionary biology (Rieseberg *et al.* 2002). Accordingly, the genetic basis and evolution of quantitative variation are receiving renewed attention (Lai *et al.* 2007; Barton and De Vladar 2009; Chang and Noor 2007). Nonetheless, most studies have not exploited information about quantitative trait locus (QTL) effects to infer selective histories (but see Rieseberg *et al.* 2002; Lexer *et al.* 2005; Albertson *et al.* 2003).

An innovative attempt (Orr 1998) to integrate QTL data and population genetic theory generated two tests for historical selection based on the proposition that selection generates a preponderance of QTL effects in the same direction (True *et al.* 1997). One test, the QTL Sign Test with Equal Effect (QTLST-EE), rejects the hypothesis of neutrality if more + alleles are observed than would be expected by chance. This simple test can be applied in cases of low or zero QTL effect variance. However, it has been criticized for its high false-positive rate (Anderson and Slatkin 2003). The other test, the QTL Sign Test (QTLST), attempts to compensate for ascertainment bias in the QTLST-EE by conditioning not just on a difference having been observed between the two lines but by the full observed difference having been observed. Orr argued that tests for selection are more likely to be performed on traits that exhibit a large phenotypic difference and that having observed a large phenotypic difference, we are more likely to detect an excess of + alleles. To this end, the QTLST conditions on the phenotypic difference by asking whether there is a preponderance of + alleles compared with randomly assigned QTL effects that result in a phenotypic difference at least as large as that observed. Anderson and Slatkin (2003) showed that the QTLST indeed controls for false-positives caused by trait ascertainment but did not examine the true-positive rate based on levels of selection and the variance of QTL effects.

Conditioning on the observed phenotypic difference has the consequence that the gross phenotypic difference itself has no effect on the inference of selection. In contrast, because selection acts directly on phenotype, phenotypic divergence is generally touted as evidence in favor of selection. Accordingly, in approaches testing for statistically significant evolution of phenotype by natural selection, gross phenotypic difference has historically been precisely the signal assessed (Lande 1976, 1977; Lande and Arnold, 1983; Lynch 1990; Turelli *et al.* 1988; Charlesworth 1984). With the observed phenotypic difference conditioned away by the QTLST, the test would appear to be likely to exhibit very low power; yet it can still yield positive results on some data sets (Lexer *et al.* 2005). Here we first present an analytical example demonstrating the negligible power of the QTLST for data sets with low QTL effect variance. Second, we show that its power depends peculiarly on the variance of QTL effects. To demonstrate this, we simulate QTL data under a model of selection and use the simulated data to assess the test's sensitivity to selection. We characterize how the QTLST performs in comparison with a sign test by setting aside the information regarding the number of allelic effects in each direction and assessing whether the QTLST is more likely to detect selection under more selective conditions.

## Analysis

Conceptually, a test of neutrality based on QTL effects should reject neutrality whenever presented with a sufficient number of loci whose effect directions are aligned with the difference between lineages. In this section, we analytically evaluate the QTLST in the “equal effects” case when all QTL effects have the same sign and magnitude. We show that the hypothesis of neutral evolution remains as far from being rejected as possible, irrespective of the number of + alleles, a point that is not obvious in the original presentation of the test.

Suppose that a trait is controlled by a number of loci (for example, 10), each of which may have + or − alleles, all of equal magnitude. Assume further that the trait is under such strong selection for enlargement that every QTL locus acquires the + substitution.

From Orr (1998), the calculation of the QTLST *P*-value for rejection of neutrality is

(1) $$P=\sum_{i=n_{+obs}}^{n}\text{Pr}\{n_{+}=i|2\sum G_{1}\geq R\},$$

where *i* is an index variable, *n* is the number of QTL loci observed, *n_+obs_* is the number of + alleles observed, *R* is the actual observed phenotypic difference between populations, and 2∑***G***_1_ is the phenotypic difference obtained by resampling from the observed distribution of QTL effects between the populations. The vector ***G***_1_ comprises the allelic effect values of the loci in the first of the two populations. In this model, the effects are additive. The two before the summation accounts for the fact that the high line allelic values are represented as ***G***_1_ and the low line values as −***G***_1_. With 10 loci,

(2) $$P=\sum_{i=n_{+obs}}^{10}Pr\left\{n_{+}=i|2\sum G_{1}\geq R\right\}.$$

Because in this example all 10 loci have a + allele in the “selected” population,

(3) $$P=Pr\{n_{+}=10|2\sum G_{1}\geq R\}.$$

Because all loci have equal effect, there is only way to sample from the distribution and assign + or − alleles to each locus that results in a phenotypic difference between populations that is (a) in the correct direction and (b) as large as that observed. That way is for all 10 loci to have + alleles, so that 2∑**G**_1_ = *R*. Thus, the proportion of times that the observed number of + loci (*n*_+_) is 10 when 2∑**G**_1_ ≥ *R* is 1.0. Therefore, *P* = 1.0 ≫ 0.05, and thus, the hypothesis of neutral evolution is as far from being rejected as is quantitatively possible. In fact, using this test, one fails to reject the null hypothesis of neutrality when effects are of equal size and in the same direction, irrespective of how many + alleles, are observed.

Although this case of exactly equal effect sizes is not very likely, it serves two important purposes. First, it illustrates the loss of power inherent in the QTLST's conditioning on the phenotypic difference. In this extreme case, conditioning on this difference throws out the entire signal left by selection, leaving the test nothing to operate on. Second, because it associates zero power with zero variance in effect sizes, it implies that rejection of neutrality would become increasingly probable with increased variance of QTL effects. In contrast, Miller *et al.* (2006) demonstrated that the response to selection of a quantitative trait does not depend on this variance when mutation is negligible. The following section tests this sensitivity of the QTLST to the variance of QTL effects by simulation.

## Simulations

To determine generally whether the QTLST accurately and precisely detects selection, we mapped the probability of fixation to the level of selection on + or – alleles in the two lineages. We performed the mapping via a model of QTL evolution under selection that encompasses the original QTLST's putatively neutral model. For *n* loci, presuming no directionality to mutation,

(4) $$Pr\{k+\text{alleles fix|neutral model}\}=\left(\begin{array}{c} n\\ k \end{array}\right){\left(\frac{1}{2}\right)}^{k}{\left(\frac{1}{2}\right)}^{n-k}.$$

A conceptually linked selective model should reduce to this case when *s*, the selection coefficient, is 0, such that the

(5) $$Pr\{k+\text{alleles fix|selective model}\}=\left(\begin{array}{c} n\\ k \end{array}\right){\left(\pi \left(s\right)\right)}^{k}{\left(1-\pi \left(s\right)\right)}^{n-k},$$

where $\pi \left(s\right)\to \frac{1}{2}$ as s→0.

To characterize the probabilities of fixation given selection coefficients, we applied a Markov model of allelic state with transition probabilities characterized by the solution of selection-diffusion equations (Kimura, 1962; Bedford and Hartl 2009). Based on the Markov model, equilibrium solutions can be straightforwardly derived for the proportion of + alleles (see *Appendix*). Substituting Equation A4 from the *Appendix* for π(s) in Equation 5 yields

(6) $$Pr\{k+\text{alleles fix}\}=\left(\begin{array}{c} n\\ k \end{array}\right){\left(\frac{e^{4Ns}}{e^{4Ns}+e^{2s}}\right)}^{k}{\left(\frac{e^{2s}}{e^{4Ns}+e^{2s}}\right)}^{n-k},$$

where *N* is the population size.

To determine the relationships between the power of the QTLST, the false-positive rate of the QTLST, and the variance of QTL effects, we simulated QTL data (*n* = 10) by binomial sampling (Equation 6). We assigned a suite of selection coefficients that, in the context of our model of selection with N = 10^6^, produce fixation probabilities spanning from 0.5 (neutrality) to just below 1 (nearly assured fixation; Table 1). Our suite of selection coefficients maps to the full range of probabilities of fixation because selection coefficients larger than *s* = 2 × 10^−6^ under our model would produce asymptotically smaller increases in the probability of fixation and thus would generate results essentially equivalent to *s* = 2 × 10^−6^. We drew allelic effect sizes from a flexible empirically and theoretically supported gamma distribution (Orr 1998, 1999, 2003). The shape and scale parameters of the gamma distribution were set equal to each other to explore variances ranging from 0.06 to 3.8 while maintaining the same mean. We then applied both the QTLST and the QTLST-EE to 10,000 sets of simulated QTL for each value of *s* and variance of allelic effects, preserving the original C code for the QTLST from Orr (1998).

**Table 1:** Probabilities of being in the selected (+) allelic state given a range of selection coefficients and a population size (*N*) of 10^6^ (Equation A4)

Selection Coefficient	Probability of Selected Allelic State
0.0	0.50
1.25 × 10^−7^	0.62
2.5 × 10^−7^	0.73
5.0 × 10^−7^	0.88
1.0 × 10^−6^	0.98
2.0 × 10^−6^	0.9997

Selection coefficients used to simulate QTL data were chosen to span the full range of probabilities of being in the selected state, given a population size of 10^6^. At one extreme, very small selection coefficients will result in virtually no difference from the equal probabilities of allelic state that correspond to the neutral model (Equation 4). At the other extreme, large selection coefficients will result in virtually no difference in the relative probability of fixation from the certain fixation of the selected state that corresponds to infinitely strong selection.

We first simulated effect sizes and directions assuming that selection coefficient was independent of QTL effect magnitude. However, it may be more realistic to assume that the selective value is proportional to the phenotypic effect (Lande 1976). Introducing a correlation between QTL effect size and allelic state could affect the power of the test. To assess this effect, we also applied both tests to simulated QTL data in which the selection coefficient used to calculate the probability that a given locus had a + allele was

(7) $$s\prime =\left(\frac{z}{z¯}\right)s,$$

where *s′* is the selection coefficient used to calculate the probability of fixation, *z* is the phenotypic effect drawn from the gamma distribution, $\overset{¯}{z}$ is the mean effect, and *s* is a selection coefficient from Table 1.

## Results

**Figure 1:**
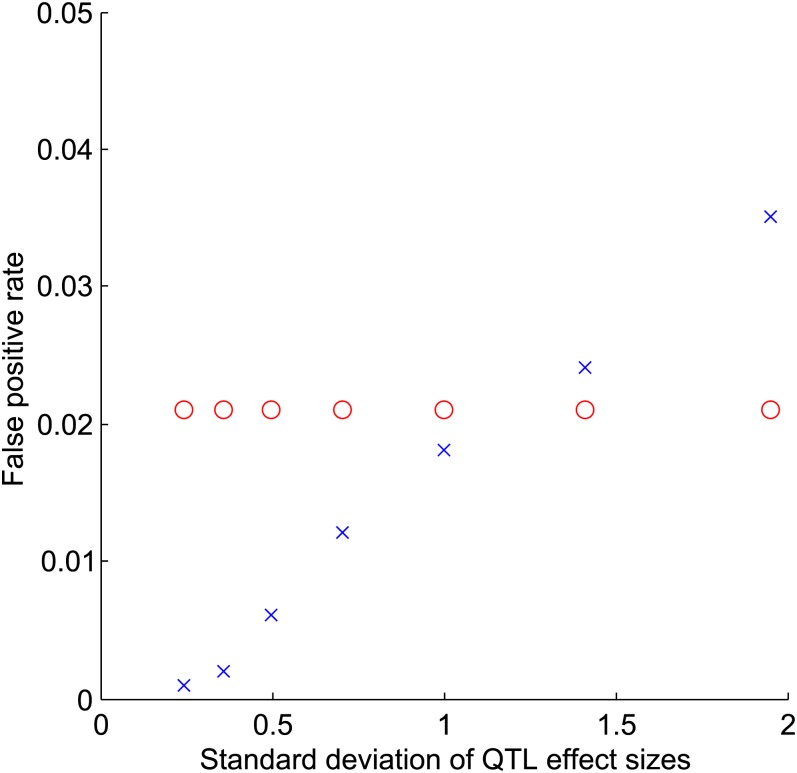
False-positive rates *vs.* standard deviation of QTL effect sizes. Circles represent the QTLST-EE, and crosses represent the QTLST.

When applied to simulated QTL data, the QTLST-EE exhibited a false-positive rate of 0.021 across all variances, whereas the false-positive rate of the QTLST rose from 0.001 when the standard deviation of effect sizes was 0.24 to 0.035 when the standard deviation was 1.95 (Figure 1).

**Figure 2:**
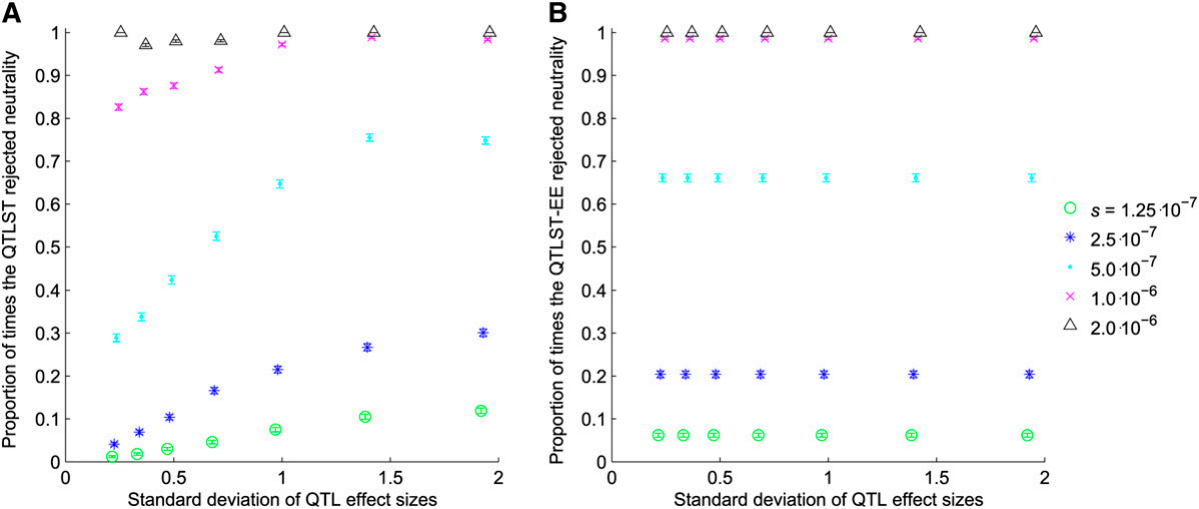
Proportion of samples for which tests rejected neutrality *vs.* the standard deviation of QTL effect sizes when selection is independent of effect size. (A) Proportion of rejections of neutrality by the QTLST. (B) Proportion of rejections of neutrality by the QTLST-EE.

In the simulations where the strength of selection was independent of the effect size of the mutation, the QTLST detected selection more often when the variance of effect sizes was high than when the variance was low for all levels of selection except *s* = 2 × 10^−6^, when there was little discriminating power because it nearly always detected selection (Figure 2A). In contrast, the QTLST-EE showed no sensitivity to the variance of QTL effects (Figure 2B).

**Figure 3:**
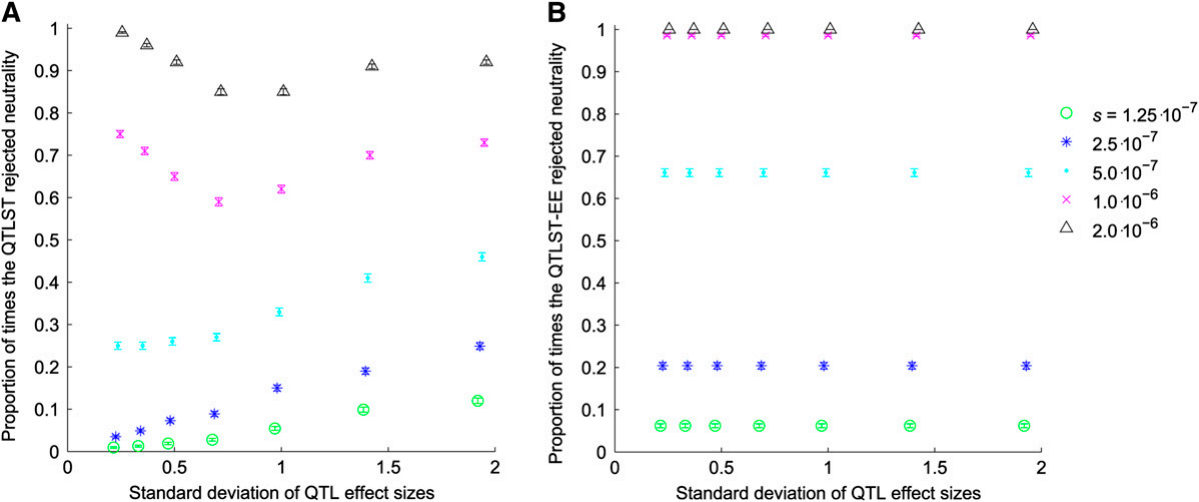
Proportion of samples for which tests rejected neutrality *vs.* the standard deviation of QTL effect sizes when selection is proportional to effect size. (A) Proportion of rejections of neutrality by the QTLST. (B) Proportion of rejections of neutrality by the QTLST-EE.

In the simulations where the strength of selection was proportional to the effect size, the QTLST detected selection less often in lower variance QTL samples for *s* < 10^−6^ and less often for intermediate variance samples for *s* ≥ 10^−6^ (Figure 3A). The QTLST-EE showed little dependence on variance of effect sizes for *s* < 5 × 10^−7^ but detected selection less often in higher variance samples for *s* ≥ 5 × 10^−7^ (Figure 3B).

**Figure 4:**
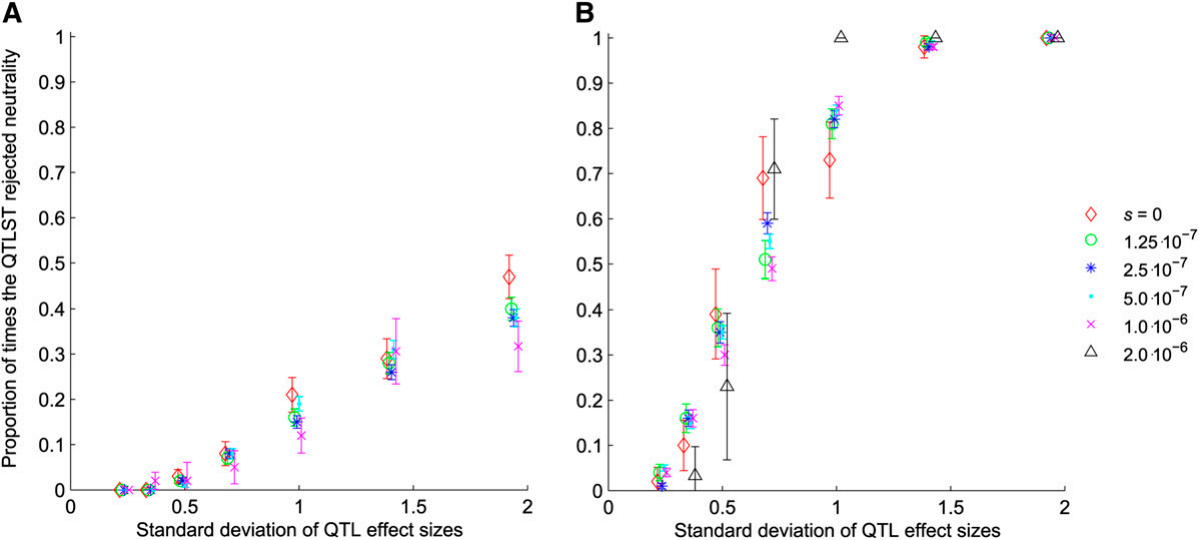
Proportion of samples for which QTLST rejected neutrality *vs.* the standard deviation of QTL effect sizes when selection is independent of effect size, broken down by number of + alleles detected. (A) Proportion of rejection when 8 out of 10 + alleles were detected. (B) Proportion of rejection when 9 out of 10 + alleles were detected. When fewer than 8 + alleles were found, the test never rejected neutrality. When 10 + alleles were found, the test always rejected neutrality. When *s* was set to a value of 2^*^10^−6^, there were never fewer than 9 + alleles in our simulations.

To better characterize these results, we plotted the results of the QTLST segregated by the number of + QTL detected. In the simulations where selection was independent of effect size, for a given number of + alleles, frequency of rejection increased with increasing variance of the distribution of effect sizes, but it did not vary with selection coefficient (Figure 4). Accordingly, for each level of variance, the proportion of times the test rejected neutrality was approximately equal for all selection coefficients, including *s* = 0. This independence of the selection coefficient to rejection of neutrality manifested stochastically for 8 observed + QTL (Figure 4A), and for 9 observed + QTL (Figure 4B). For all other numbers of observed + QTL, the proportion of times the test rejected neutrality was exactly equal for all selection coefficients, including *s* = 0: when fewer than 8 + QTL were observed, the test never rejected neutrality, and when 10 + QTL were observed the test always rejected neutrality, regardless of the selection coefficient and variance of QTL effects.

**Figure 5:**
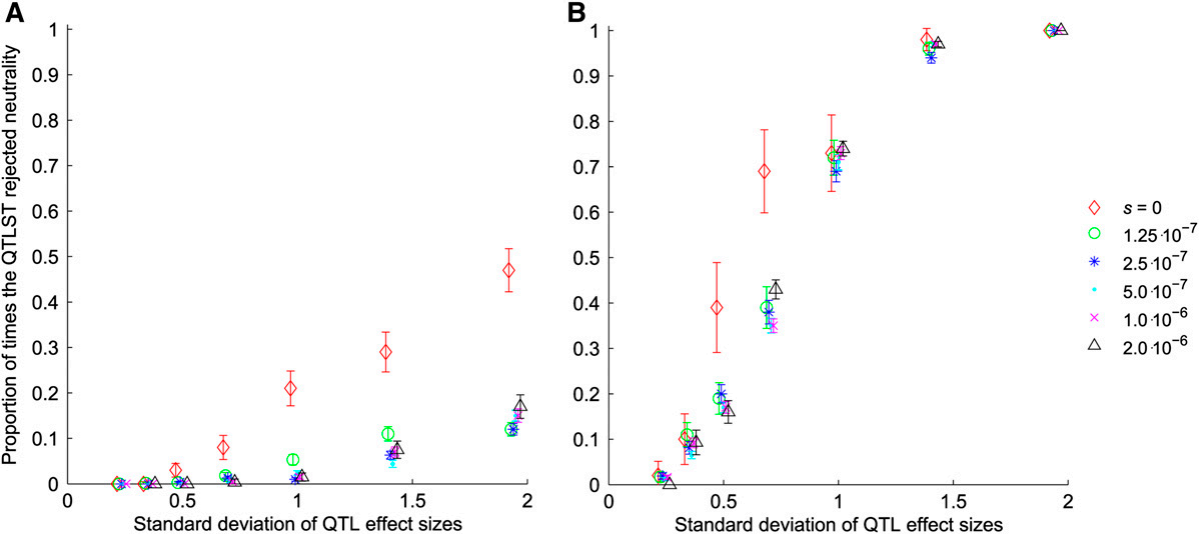
Proportion of samples for which QTLST rejected neutrality *vs.* the standard deviation of QTL effect sizes when selection is proportional to effect size. (A) Proportion of rejection when 8 out of 10 + alleles were detected. (B) Proportion of rejection when 9 out of 10 + alleles were detected. When fewer than 8 + alleles were found, the test never rejected neutrality. When 10 + alleles were found, the test always rejected neutrality.

When strength of selection was proportional to the effect size of mutations, for a fixed number of + alleles, frequency of rejection again increased with the variance of the distribution of effect sizes, but it was again largely independent of selection coefficient (Figure 5). This independence of selection coefficient manifested stochastically when 8 (Figure 5A) or 9 (Figure 5B) + alleles were observed. For both 8 and 9 + QTL with proportionality of the selection coefficient, *s* = 0 was more likely to lead to a conclusion that selection had been in operation than other selection coefficients, which were all otherwise equivalent. Simulations of selection coefficients smaller than 10^−7^ showed increasing probability of rejecting neutrality with decreasing selection coefficients, reaching a plateau at the probability for *s* = 0. As in the case with no proportionality of selection, when fewer than 8 + alleles were observed, the test never rejected neutrality, and when 10 + alleles were observed, the test always rejected neutrality.

## Discussion

We have shown that the QTLST is highly sensitive to the variance in QTL effects. Furthermore, our simulations demonstrate that for a given number of observed + QTL, the QTLST does not reject neutrality more often when the QTL it is applied to are generated under stronger selection (Figures 4 and 5). Instead, for a given number of observed + QTL, the probability of rejecting neutrality is independent of the selection coefficient used to generate the QTL (Figure 4). In fact, once the number of + QTL is set, the power of the test to reject neutrality is determined by the variance in effect size among the QTL, a factor which we have shown has no bearing on whether the test should detect selection.

When selection was proportional to effect size, the QTLST was more likely to reject neutrality when *s* = 0 than when *s* > 0 (Figure 5). This effect results from conditioning on the phenotypic difference. Under proportional selection, + alleles are more likely to fix at large-effect loci than at small effect loci. Therefore, we expect greater phenotypic differences in selected traits than in neutrally evolving traits with the same number of + alleles fixed. Under the test's null model, large numbers of + alleles are less likely given a small phenotypic difference than a large phenotypic difference. Thus, conditioning on the trait difference leads to smaller *P*-values and more frequent rejection of neutrality when *s* = 0.

The QTLST is, appropriately, more likely to conclude that selection has occurred when there are more + alleles; thus, it does have some power to detect selection (Figures 2 and 3). This power arises from the fact that resampling from a larger number of alleles with significant variance in effect is more likely to exceed the observed phenotypic difference. However, this power is causally unrelated to the fact that + alleles fix more frequently under positive selection than they do under neutrality. Accordingly, the QTLST yields plausible results when one examines the results comparing across the number of + QTL (Figures 2 and 3), but within a given number of + QTL, its results are strongly dependent on the variance of QTL effects (Figures 4 and 5). In fact, for QTL data sets with low variance of effect sizes, the power to detect selection asymptotes to zero. Accordingly, the false-positive rate of the QTLST also depends on the variance of effect sizes (Figure 1). For QTL samples with large variance, the QTLST has a higher false-positive rate than the QTLST-EE. For small-variance samples, the QTLST has a smaller false-positive rate than the QTLST-EE, but it also has a correspondingly low true-positive rate. Thus, the low false-positive rate arises at the expense of power. Note that the QTLST-EE's false-positive rate is less than 0.05 because the binomial distribution is discrete: with 10 QTL the test rejects neutrality when 9 + QTL are observed (*P* = 0.021) but not when 8 + QTL are observed (*P* = 0.11). Nine or more + QTL were observed 0.021 of the time in our neutral simulations, so that is the false-positive rate.

The details of the simple model of selection that we employ are irrelevant to these conclusions. The purpose of the precise selective model in our simulations is only to establish a correspondence between strength of selection and the probability of the + allele fixing. For all plausible models of directional selection it will be true that (1) as selection increases, the probability of the QTL having the + allele goes to 1, and (2) for neutral traits, the probabilities of having the + and – alleles are equal. Our model of selection allowed us to define a range of selective strengths that generated probabilities of having the + allele ranging from 0.5 to slightly less than 1, encompassing the entire relevant range. Had we chosen to use a different model of selection, we would then have tested somewhat different selection coefficients to map to the same range of fixation probabilities, but the results of the simulations would have been identical.

In conclusion, both the QTLST and the QTLST-EE are problematic when applied to certain types of data. Anderson and Slatkin (2003) previously demonstrated that the QTLST-EE suffers from ascertainment bias when QTL data sets are selected for testing based on their large phenotypic differences. However, in attempting to correct for this bias, the resampling procedure of the QTLST introduces sensitivity to the variance of the QTL effects, a result which has no basis in the history of neutrality or selection. In fact, for the extreme case of zero variance, the test has no power at all. Therefore, researchers using these tests must carefully consider not only the possible ascertainment bias in their data but also the variance of QTL effects. A recent alternative approach to testing for selection with QTL data (Rice and Townsend 2012) avoids these issues by capitalizing on information about mutation effect distributions to construct more realistic neutral and selective models.Can't read 3B2 tag because stream don't exist.Tag: Eq_1

## Appendix

### Modeling Selection

To match the neutral model underlying the QTLST, we draw QTL allelic effect sizes from a distribution, and only two final states for the parental lineages are allowed: homozygous + and homozygous -. For the sake of intuitive clarity, we assume that the + allele is the one selected for, though of course in natural systems selection may favor phenotypes divergent in either direction. We assume that one of these two states is the ancestral state, that the other is a derived state, and that the mutation responsible is reversible.

To characterize the outcome of a history of selection, we calculate the distribution of + and - alleles by drawing from the equilibrium probabilities of state according to a Markov model with transition probabilities characterized by the solution of selection-diffusion equations. Starting with an initial population fixed for the - allelic state and assuming that the diffusion equation results from Kimura (1962) hold, we find that,

(A1) $$\text{Pr}\left\{\text{fixation of}+\text{allele}\right\}=\frac{1-e^{-2s}}{1-e^{-4Ns}},$$

and

(A2) $$\text{Pr}\left\{\text{fixation of-allele}\right\}=\frac{1-e^{2s}}{1-e^{4Ns}}.$$

To solve for the equilibrium probability of the presence of expansive alleles, one must calculate the rates of transitions between the allelic states.

Let *μ* be the rate at which mutations that affect the trait arise. Then, the rate of beneficial transition from the - allele to the + allele is *Nμ* times the fixation probability of the + allele (Equation A1). Similarly, the rate of deleterious transition from the + allelic state to the - allelic state is *Nμ* times the fixation probability of the – allele (Equation A2). Solution of the equilibrium state probabilities from these rates of transition yields that the

(A3) $$\text{Pr}\left\{+\text{allele}\right\}=\frac{2N\mu \left(\frac{1-e^{-2s}}{1-e^{-4Ns}}\right)}{2N\mu \left(\frac{1-e^{-2s}}{1-e^{-4Ns}}\right)+2N\mu \left(\frac{1-e^{2s}}{1-e^{4Ns}}\right)}.$$

Further algebra simplifies Equations A3 to

(A4) $$\text{Pr}\left\{+\text{allele}\right\}=\frac{e^{4Ns}}{e^{4Ns}+e^{2s}}$$

Substituting Equation A4 for *π*(*s*) in Equation 5 yields Equation 6 in the main text. Note that these equations are independent of the rate of mutation of the trait.

Kimura's equations (Equations A1 and A2) do not apply exactly in the case of multiple competing alleles. Nevertheless, a consistent relation between selection coefficient and probability of fixation does, and the result relevant to our analysis, the ratio of the fixation rates of the expansive and diminutive alleles, should not be adversely affected.
